# Supplementary material for: Modeling glioblastoma heterogeneity as a dynamic network of cell states
Source: Mol Syst Biol. 2021 Sep 16;17(9):e10105. doi: 10.15252/msb.202010105 (PMC8444284; doi:10.15252/msb.202010105)
Supplement: Supplementary file 6 — Source Data for Figure 5 [file MSB-17-e10105-s004.zip › Figure5A_sourcedata/GSEA_3071/nosigdb_stateVI.GseaPreranked.1622795169056/ROSEN_KRONA_TI_UNPUBL.html]

Details for gene set ROSEN\_KRONA\_TI\_UNPUBL[GSEA]

|  || Dataset | state63071 |
| Phenotype | NoPhenotypeAvailable |
| Upregulated in class | na\_pos |
| GeneSet | ROSEN\_KRONA\_TI\_UNPUBL |
| Enrichment Score (ES) | 0.23153459 |
| Normalized Enrichment Score (NES) | 1.3073045 |
| Nominal p-value | 0.13777778 |
| FDR q-value | 0.19134684 |
| FWER p-Value | 0.952 |
Table: GSEA Results Summary

  

Fig 1: Enrichment plot: ROSEN\_KRONA\_TI\_UNPUBL      
 Profile of the Running ES Score & Positions of GeneSet Members on the Rank Ordered List

  

| PROBE | GENE SYMBOL | GENE\_TITLE | RANK IN GENE LIST | RANK METRIC SCORE | RUNNING ES | CORE ENRICHMENT || 1 | FOXS1 |  |  | 37 | 0.925 | 0.0431 | Yes |
| 2 | KCTD9 |  |  | 115 | 0.712 | 0.0628 | Yes |
| 3 | ANKRD1 |  |  | 137 | 0.684 | 0.0965 | Yes |
| 4 | SPHK1 |  |  | 141 | 0.677 | 0.1347 | Yes |
| 5 | AOX1 |  |  | 182 | 0.619 | 0.1593 | Yes |
| 6 | RPS17 |  |  | 252 | 0.562 | 0.1725 | Yes |
| 7 | AC016831.1 |  |  | 266 | 0.556 | 0.2010 | Yes |
| 8 | AC016831.7 |  |  | 315 | 0.532 | 0.2183 | Yes |
| 9 | CCT2 |  |  | 441 | 0.474 | 0.2110 | Yes |
| 10 | SMTN |  |  | 498 | 0.455 | 0.2217 | Yes |
| 11 | ALPL |  |  | 613 | 0.422 | 0.2143 | Yes |
| 12 | HSPG2 |  |  | 688 | 0.401 | 0.2169 | Yes |
| 13 | IL7R |  |  | 718 | 0.392 | 0.2315 | Yes |
| 14 | TRABD2A |  |  | 984 | 0.348 | 0.1781 | No |
| 15 | SORD |  |  | 1239 | 0.311 | 0.1255 | No |
| 16 | HGS |  |  | 1375 | 0.294 | 0.1050 | No |
| 17 | NOC4L |  |  | 1380 | 0.294 | 0.1208 | No |
| 18 | LINC00944 |  |  | 1481 | 0.282 | 0.1093 | No |
| 19 | DSE |  |  | 1484 | 0.282 | 0.1250 | No |
| 20 | PLCB2 |  |  | 1655 | 0.264 | 0.0931 | No |
| 21 | RPL21 |  |  | 1725 | 0.258 | 0.0888 | No |
| 22 | ISG20 |  |  | 1756 | 0.255 | 0.0952 | No |
| 23 | CSPG4 |  |  | 1916 | -0.266 | 0.0664 | No |
| 24 | ING1 |  |  | 1961 | -0.272 | 0.0699 | No |
| 25 | YEATS4 |  |  | 2054 | -0.287 | 0.0610 | No |
| 26 | UBALD2 |  |  | 2209 | -0.316 | 0.0364 | No |
| 27 | ALG6 |  |  | 2401 | -0.353 | 0.0038 | No |
| 28 | PIP5K1B |  |  | 2506 | -0.379 | -0.0032 | No |
| 29 | TSPAN31 |  |  | 2535 | -0.385 | 0.0112 | No |
| 30 | CHRDL1 |  |  | 2735 | -0.434 | -0.0190 | No |
| 31 | ITIH5 |  |  | 2874 | -0.485 | -0.0293 | No |
| 32 | ARL4D |  |  | 3076 | -0.577 | -0.0518 | No |
| 33 | ERBB3 |  |  | 3116 | -0.596 | -0.0282 | No |
| 34 | ITM2C |  |  | 3187 | -0.652 | -0.0101 | No |
| 35 | HIST1H1E |  |  | 3504 | -1.125 | -0.0328 | No |
| 36 | PLEKHA5 |  |  | 3525 | -1.209 | 0.0314 | No |
Table: GSEA details [plain text format]

  

Fig 2: ROSEN\_KRONA\_TI\_UNPUBL: Random ES distribution      
 Gene set null distribution of ES for **ROSEN\_KRONA\_TI\_UNPUBL**
